# IoT based automated early detection and real time environmental monitoring for poultry farming in developing countries

**DOI:** 10.1371/journal.pone.0356528

**Published:** 2026-09-15

**Authors:** Md. Nahidul Alam, Md. Zahid Hasan Buiyan, Ratul Sarker, Imran Hossain, MD. Mehedi Hasan

**Affiliations:** Department of Electrical and Electronic Engineering, Bangladesh Army International University of Science and Technology, Cumilla, Bangladesh; North-Caucasus Federal University - Pyatigorsk Campus: Severo-Kavkazskij federal'nyj universitet Patigorskij institut filial, RUSSIAN FEDERATION

## Abstract

Poultry farming is a rapidly growing agricultural subsector worldwide and plays a significant role in meeting global demand for human protein. In Bangladesh, poultry farming is in developing phase, but it faces major challenges. Where improper temperatures, humidity, gas concentrations, dust levels, and poor ventilation significantly reduce poultry health, egg production, and overall farm profitability. To address these issues, monitoring systems are increasingly being adopted for early detection and continuous assessment of the farm environment. However, in a traditional monitoring system, the process is slow and inaccurate, and it cannot provide early warning of harmful conditions. The conventional method for poultry farming involves manual monitoring, leading to poor management and low efficiency. Smart poultry farming using Internet of Things (IoT) technology can help improve efficiency, poultry well-being, and management practices. This paper presents an IoT based early detection, monitoring and controlling system to maintain an ideal environment in a poultry farm using real time data. The testing was conducted at a poultry farm in Bangladesh; for consistency, a rigorous 15-day test was conducted. The results from the prototype system shows significant results than those from the reference sensors. For each sensor used on the prototype system, a linear correlation analysis was conducted. This confirms the system’s accuracy in measuring temperature, humidity, ammonia, and methane. To maintain the farm’s temperature, an automated cooling system using a honeycomb is also incorporated. For the microcontroller unit, the ESP32 was used, and for the IoT base platform, ThingSpeak is utilized. After performing the cost analysis of the system, it was found that the system with the early monitoring feature was constructed at a total cost of United States Dollar (USD) 43, which is convenient for developing countries like Bangladesh. The source code of the proposed system is available on GitHub.

## 1. Introduction

Rearing domesticated birds such as chickens, ducks, turkeys, and quails for food, eggs, and other products is essential for food security and economic development worldwide [1,2]. Containment systems for controlled environments are standard in a poultry farm. These may be small backyard farms or massive commercial centers. The overall objective is to keep the birds housed, fed, and monitored to attain maximum growth, reproduction, and good health [3]. Poultry farms include housing systems, feeding techniques, waste management, and biosecurity measures to reduce disease and environmental stressors. Finally, Low-cost protein is offered to enhance human nutrition [4,5]. As a scalable and flexible business, poultry farming sustains the lives of millions of people, particularly in rural areas. It is a sector that responds to the fact that the popularity of animal-based proteins will continue to rise, and the population will reach 9.7 billion people by 2050 [6,7]. The poultry industry has been expanding rapidly worldwide, turning poultry farming into a multibillion-dollar venture. It is estimated to grow to 423 billion dollars by 2025, with a compound annual growth rate (CAGR) of 7 percent since the beginning of the 21st century [8]. Urbanization, rising incomes, and a shift in diet toward intensive consumption of high protein, low cholesterol meats contribute to this growth. Annual per capita chicken consumption is 315 million tonnes worldwide (72% of total meat consumption) [9,10].The most dominant manufacturers are the United States, which handles 20 million tonnes annually; China, which handles 22 million tonnes; and Brazil, which handles 14 million tonnes. This has made them more efficient and led to vertical integration through hatcheries, feed mills, and processing, resulting in more exports. The latest technologies, such as precision feeding and automated ventilation, have lowered the feed conversion ratio (FCR) to 1.5–2.0, improving sustainability in the face of climate challenges [11]. Nonetheless, the inequality persists, as the average output is forty percent in Asia and five percent in Africa, both areas being low resource endowed [4,5].

The poultry sector in Bangladesh is developing rapidly, in 1993–94, there were 43,589 backyard poultry farms, but now there are more than 300,000 commercial farms. This is now generating 1.8 million tonnes of meat and 15 billion eggs per annum at 1.5–2% GDP, and it employs 2.5 million workers, predominantly women and young rural workers [2,4]. The industry supplies 22–27 percent of the nation’s total animal protein demand, and broilers are the main product due to the high urban population in cities such as Dhaka [1]. Since 2010, pressures on inputs have been reduced by half through government initiatives such as the National Poultry Development Policy and the verticalization of companies, including Kazi Farms, to ensure self-sufficiency. Although these improvements have been made, the industry significantly contributes to poverty reduction, and 80 percent of the farms (smallholders) augment BDT 50,000–100,000 each year. Nonetheless, some weaknesses are revealed by issues like avian influenza outbreaks, such as the loss of HPAI in 2008 [8]. The traditional methods of rearing poultry are usually either cleaning their backs over open fields (80–90% in developing countries) or using intensive systems in poultry farms. Such exercises are usually related to periodic monitoring of environmental conditions (e.g., temperature with thermometers, humidity with hygrometers) and to work intensive activities such as feeding chickens, collecting, and incubating eggs [12]. Evaporative cooling and fans are popular in the Asian and African regions to control the heat. Maize soy blends represent the feed formulas, which are 60–70% of total costs [13]. Biosecurity is based on the visual checks, quarantines, and reactive inoculations (e.g., against Newcastle disease) rather than prevention strategies [14]. Although such cost-effective approaches are suitable for smallholders, An FCR of 2.5–3.0 is observed, while seasonal variations lead to a 10–20% mortality rate.

The future of IoT based poultry farming is expected to shift toward sensor fusion, AI, and edge computing, with a 25 percent annual increase, adding 25 percent to the market, which will be worth 400 million dollars by 2030 [6,7,10]. A combination of developing computer vision (CV) and machine learning to track behavior achieves 91.78% accuracy and will assist in the early detection of diseases [8,14]. Technologies such as solar hybrids and blockchain can be used to share data from off grid systems safely [5,13,15]. It is promising scalability in Asia, as reflected in Thai EVAP-WSN hybrid pilot projects and the expectation of targeting an incremental increase of 100 billion in regional Gross domestic product (GDP) by 2030 across Asia [4,7,9]. Against the background of such developments, numerous challenges this industry faces, including expensive feed (60%−70% of total costs), disease epidemics, and environmental pressures such as heatwaves [1,2]. 90% of Danish farmers predict these problems. Poor infrastructure, including 90 percent power blackouts in rural Bangladesh, and poor digital literacy are impediments to the use of IoT. Data integrity may be undermined by cybersecurity threats, such as Distributed Denial of Service (DDoS) attacks on Wireless Sensor Network (WSNs), which can have economic impacts [7,15]. Also, a deficient policy is irreconcilable, relying on imports, and when combined with the susceptibility of climate and post-harvest losses of 10–20 percent, there are still zoonotic risks involved.

Poultry farming in developing countries like Bangladesh still depends heavily on manual and periodic monitoring, which often fails to detect dangerous environmental changes in time. Improper temperature, humidity, toxic gas accumulation, and poor ventilation are among the leading causes of bird mortality and reduced egg production. Existing low-cost IoT systems proposed in the literature have addressed some of these concerns, but most lack long-term field validation, multi-parameter sensing, or practical affordability for small-scale farmers. These persistent gaps in both practice and research make the development of a reliable, affordable, and automated monitoring solution both timely and necessary.

This study highlights an IoT based environmental monitoring system design and system integration together with field level validation of two environmentally sustainable poultry farm prototypes in Bangladesh as the primary study context. It uses an ESP32 microcontroller interfaced with integrated sensors and a ThingSpeak cloud platform to monitor seven environmental factors: temperature, humidity, ammonia, methane, dust concentration, light intensity, and litter moisture. A 15-day field trial validated sensor performance against commercially available reference instruments. Addressing these problems with IoT based solutions is a crucial aspect of risk management and of achieving sustainable, equitable growth.

The main contributions of this paper are as follows:

i. A comprehensive IoT based system integrating seven environmental sensors for simultaneous monitoring of temperature, humidity, ammonia, methane, dust, light intensity, and litter moisture offering a more holistic approach than prior studies that focus on only a few parameters.
ii. An automated early detection mechanism with real time alert capabilities (buzzer, LED, and remote notification via ThingSpeak) to enable rapid farmer response before harmful thresholds are exceeded.
iii. An automated evaporative cooling system using a honeycomb pad and fan, activated based on real time sensor data, to maintain a thermally ideal environment for poultry.
iv. A thorough 15-day field validation of prototype sensors against standard commercial reference instruments using linear correlation analysis, confirming measurement accuracy across all monitored parameters.
v. A cost-effective prototype developed at a total cost of USD 43, demonstrating practical affordability for smallholder farmers in developing countries like Bangladesh.

The uniqueness of this paper is based on the integration of various environmental sensors such as temperature, humidity, ammonia, methane, dust, light, and soil moisture to monitor and control all the critical factors that influence the health and productivity of poultry. For instance, it provides a holistic approach to poultry farm management compared to other systems that focus on a few parameters.

The following four research question was developed to understand the purpose of the study.

**RQ1:** How do sensors help to maintain an ideal environment for poultry growth?
**RQ2:** How can poultry health be improved through early detection and continuous monitoring of gases?
**RQ3:** How can automation be used to enhance poultry health, productivity, and savings?
**RQ4:** How can farmers improve disease prevention, feed intake and reproduction using historical and real time data along with predictive analytics?

To address these research issues, this study investigated the development, deployment, and verification of an IoT platform for poultry monitoring by combining sensors such as BME-680, MQ4, BH1750, water flow, dust, and capacitive soil moisture sensor and relay modules with microcontrollers such as Arduino and ESP32.

The main objective of this given paper is given below:

- An automated ventilation mechanism that operates based on real time environmental data.
- Helps the farmer detect harmful conditions early and take quick action, increasing farm efficiency.
- Reducing chicken mortality and increasing egg production.

## 2. Literature review

Monitoring the poultry industry is a pressing need in developing countries like Bangladesh, where poultry meat accounts for the majority of meat consumption. According to the Bangladesh Poultry Association, 2 billion BDT worth of birds were lost in 2024 [16]. It has been investigated that early monitoring can prevent these losses. A notable study regarding monitoring was conducted by G. Da Rocha Balthazar et al. [17]. The author presents the development and validation of an IoT-based environmental sensor designed to monitor the microclimate in broiler poultry houses. While a low-cost device was prepared, the power management issue remained unresolved, and validation was not conducted for a long term, such as 10–15 days. Additionally, O. K. Hélène et al. [18] discussed TinyML and IoT based system designed for the automated analysis and monitoring of chicken egg quality in the poultry industry. However, the system lacks full scale field deployment and limited environmental sensing. Throughout this study, an extensive investigation was conducted, leading to the inclusion of 20 papers in the summary. The document covered the years 2019–2025. Table 1 summarizes each paper’s primary focus and research contribution. By closely reviewing these works, key research gaps and limitations were identified: most papers did not conduct extensive prototype testing; few validated results against commercially available sensors; and only a limited number described stored data scenarios for one or two specific months. By addressing these particular gaps, the model has been prepared and describe it in detail in the methodology section.

**Table 1:** Summarization of recent studies for poultry monitoring system.

Ref.	Authors	Year	Major Contribution	Area of main focus	Limitations
[22]	Elwakeel et al.	2025	Demonstrated an affordable and easy way to observe farms, saving time and making the work more automated.	IoT base Automated control and monitoring system.	Limitations include GSM dependence, low-cost sensor accuracy, cross sensitivity, lack of field validation, no safeguards, and limited scalability.
[23]	Bhattad et al.	2025	An IoT based system that records the flow of gas emissions throughout the day and forecasts amounts for the following day.	IoT base real time air quality monitoring system.	Current environmental detection technologies fail to integrate complex interactions, leading to inaccurate control and predictions.
[10]	Godinho et al.	2025	IoT based smart poultry farming systems enhance production and welfare through wireless monitoring and control.	IoT base wireless farm monitoring and controlling system.	Local microclimates, sensor issues, and forecast uncertainties can hinder outdoor temperature and humidity reliance.
[24]	Qi et al.	2023	By combining information about animal needs with environmental data, the author discussed accurate and effective control systems.	Monitoring of environmental conditions in poultry industry using sensor technology.	Current environmental detection technologies fail to integrate complex interactions, leading to inaccurate control and predictions.
[25]	Chang et al.	2023	Developing a low-cost, safe, and automatic ammonia gas generation system for poultry health testing in a controlled chamber.	IoT sensors base poultry house monitoring system.	AI war rooms can almost remove downtime but are limited by data quality, model reliability, humans, integration, complexity and costs.
[26]	Lufyagila et al.	2022	Creating a profitable IoT solution for smallholder farmers with limited resources.	IoT base temperature, humidity, and ammonia level detector system.	The IoT poultry system faces scalability, cost, technical issues, and security risks, hindering adoption.
[27]	Orakwue et al.	2022	The project involves the implementation of a cost-effective IoT system for monitoring and controlling environmental parameters.	IoT base chicken farm monitoring system to reduce mortality, and boost production.	Work lacks sensor calibration, reliability, and small-scale farmer capacity.
[28]	Zheng et al.	2021	Future poultry farming will leverage IoT and cloud databases for enhanced efficiency and disease management.	An A based poultry farming system to improve efficiency and disease detection.	The system’s effectiveness may decline due to reliance on sensor networks, cloud connectivity, and accurate data, especially under poor conditions.
[29]	Hofstetter et al.	2021	Developing an IoT based system for efficient indoor poultry environment monitoring and control using cloud computing.	An ammonia gas generator was developed to control ammonia levels in poultry houses.	Increased ammonia enhances runtime and stabilization. MQ-137 sensors drift with humidity, requiring redesign for scaling.
[30]	Shahzad et al.	2021	The MEC system effectively reduces heat stress in chickens, making it ideal for hot climates like Multan.	Inexpensive, eco-friendly evaporative cooling systems are developed to reduce heat stress in poultry farms.	Does not incorporate desiccant integration, full scale testing, economics, and long-term operations.
[31]	Hambali et al.	2020	Reduce mortality and increase productivity in warm climate areas.	IoT automation enhances productivity by reducing chicken mortality and optimizing conditions.	IoT agricultural systems in rural Brunei face challenges like outages, costs, accuracy, and privacy issues.
[32]	Pereira et al.	2020	A new technological solution for agriculture and animal welfare at just 13% of the cost.	IoT based hardware and software monitor environmental conditions in chicken houses.	Validation occurred only in poultry houses, limiting applicability to other livestock due to varying conditions.
[33]	Akhund et al.	2020	Improving a system for building a sustainable, renewable energy powered IoT system for poultry farms.	IoT smart poultry farms utilize renewable energy and sensors for monitoring.	IoT poultry farming relies on stable internet, power, and maintenance. Its failures disrupt monitoring and data storage.
[34]	Lorencena et al.	2020	Creating a sensor network system for temperature control and comfort monitoring in poultry farms.	Developed a sensor base prototype for temperature control in poultry farm.	Real world deployment faces costs, complexity, integration challenges, and validation needs for poultry house retrofitting.
[35]	Astill et al.	2020	Precision Livestock Farming enhances poultry management through big data, IoT, sensors, and automation technologies.	Smart poultry management employs technology for bird welfare, enhancing feeding, monitoring, and disease detection.	Integrating PLF, big data, and IoT in poultry farming faces technical, governance, cost, and labor challenges.
[36]	Saeed et al.	2019	Strategies were developed to manage heat stress in poultry farms, enhancing welfare and productivity.	High temperatures affect chicken health and productivity; management and diet can mitigate these effects.	Heat stress threatens poultry production, impacting growth and quality. Farmers must enhance management, nutrition, and explore innovative solutions to mitigate effects.
[37]	Manshor et al.	2019	An IoT model for smart farming utilizes sensors and apps for real time monitoring and alerts.	An IoT based system for checking temperature, humidity, and power connection at any time and in any place.	Sensor drift, placement variability and environmental interference reduce accuracy.
[38]	Islam et al.	2019	GSM and IoT combine to automate poultry farm monitoring, enhancing security and environmental data management.	The IoT model monitors farm security and alerts farmers via SMS for fire and theft emergencies.	Sensor failures, connectivity issues, power outages, and costs hinder adoption in remote farming environments.
[39]	Deepika et al.	2019	Affordable poultry water monitoring and low-cost sensors enhance water quality, improving health and productivity.	To develop a water quality testing system for poultry farms.	The system’s focus on pH, turbidity, and temperature overlooks other vital contaminants impacting poultry health, like pathogens and heavy metals.
[40]	Ranjan et al.	2019	Heat affects poultry behavior, health, and production; solutions include improved ventilation, nutrition, and housing.	High temperatures and humidity harm chickens, egg production; ventilation and electrolytes mitigate economic losses.	The review focuses only on broilers and laying hens in hot climates and does not address contemporary technologies such as IoT based monitoring or automated ventilation systems.
This Paper			An automated early-detection system with real-time environmental data analysis. As chicken health and egg production depend on the heat effects, proper ventilation with temperature and humidity control is included in the solution. User-friendly data visualization from anywhere, as it is an early detection system, so a huge farm loss can be avoided.	Automated environmental monitoring with an early detection system and proper ventilation with an integrated solar panel for continuous power supply.	Integration of mobile application alerts and additional remote tracking features to further increase usability and robustness in diverse farm contexts. Additionally, the MQ series gas sensors used in the prototype require periodic recalibration, as prolonged exposure to humidity and temperature fluctuations can gradually affect their measurement accuracy.

Apart from poultry farming, IoT sensor technologies have gained a large number of applications across different domains of smart agriculture. Morchid et al. [19] has proposed an IoT based early fire detection system for smart agriculture integrating smoke and flame sensors, Raspberry Pi act as gateway and ThingSpeak cloud platform is used for data visualization in real time for prevent the calamity before the risk. This idea was then built upon in a follow up [20] where the authors created a web application featuring important cybersecurity components login authentication, HTTPS protocols, etc. based on Flask so that farmers could monitor real time fire detection data directly on a web browser to improve the safety of their farms. In another related work, Morchid et al. [21] proposed an IoT based smart irrigation management system that combines embedded systems, cloud computing via the ThingsBoard platform, and multi sensor data acquisition to optimize water usage and enhance agricultural water security. All in all, these works show that IoT sensor platforms have common architectural elements such as real time monitoring, cloud interfacing, automated responses based on threshold values, and remote accessibility, that can be utilized for various agri-related use cases including poultry farm environmental monitoring, fire detection for greenhouses and even water management for crop farming.

## 3. Methodology

### 3.1. Smart poultry farming environmental control system

Field testing was conducted at a privately owned commercial poultry farm in Bangladesh with the consent of the farm owner. No institutional or governmental permit was required for this study, as the work involved only non-invasive environmental monitoring using IoT sensors and did not involve any experimentation on animals or human participants. Field access was granted by the farm owner, and no formal ethical approval from an Institutional Review Board (IRB) or ethics committee was required. This study was conducted in accordance with the ethical guidelines of Bangladesh Army International University of Science and Technology, Cumilla, Bangladesh.

**Fig 1:**
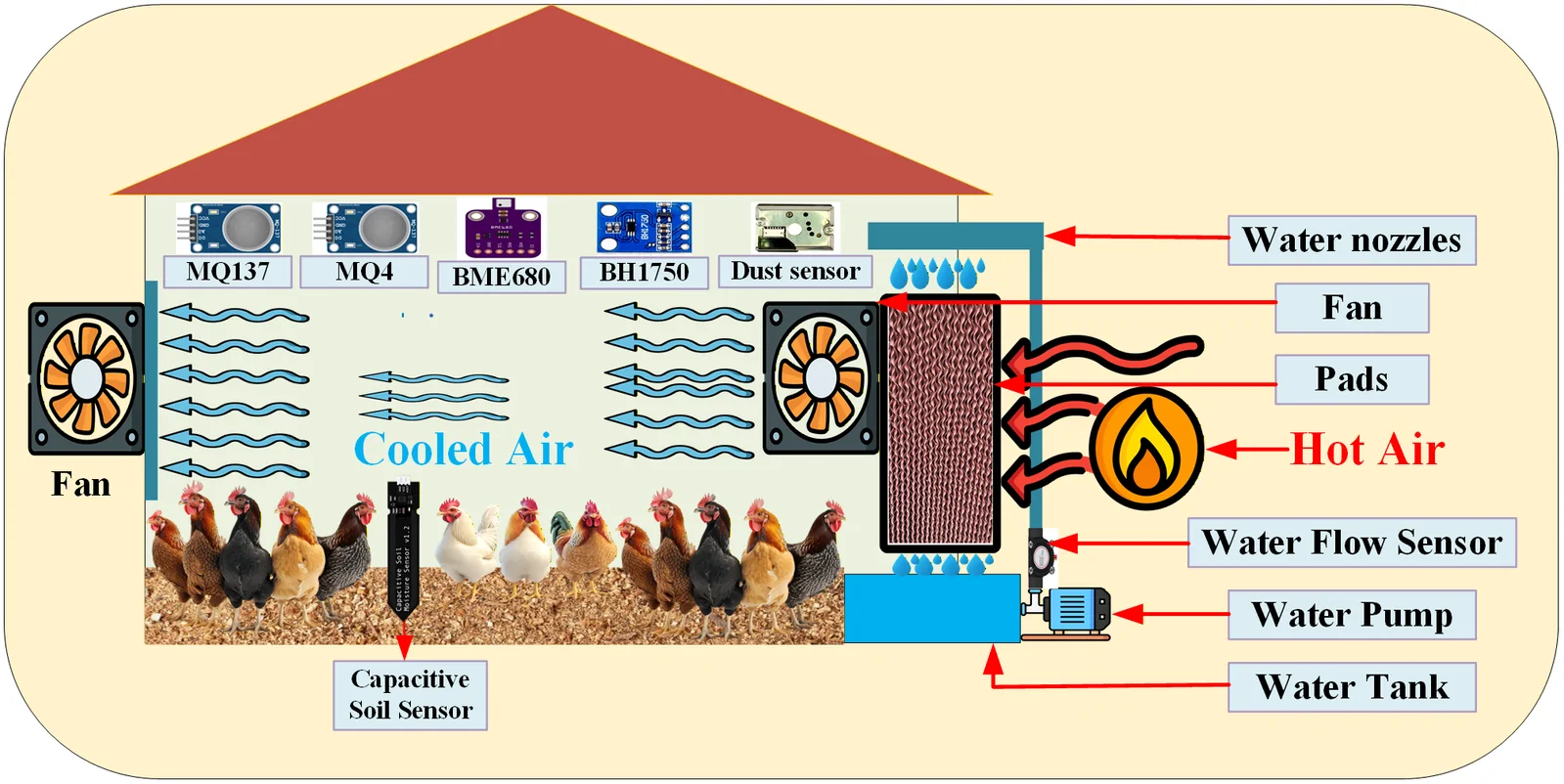
Architecture of an IoT based multi-purpose smart poultry farm.

In Fig 1, an Internet of Things (IoT) based smart poultry farming environmental control system is depicted. The system ensures the ideal state for chickens by monitoring and controlling temperature, humidity, air quality, and soil moisture. Metal Oxide gas sensor for Ammonia (MQ137) and Metal Oxide gas sensor for Methane (MQ_4_) sensors continuously monitor harmful gases, including ammonia (NH_3_), CO_2_, methane (CH_4_), and other combustible gases, which are mostly produced by poultry waste. The BME680 sensor is used in the system to monitor temperature, relative humidity, and air quality. To expand egg production and bird activity, BH1750 sensors are used to measure light intensity. Dust sensors are used to protect chickens against pulmonary diseases, and a Capacitive Soil Sensor tracks the moisture level in the litter. The heating control system uses a pad fan cooling system for efficient temperature regulation. Water is stored in a water tank. During cold temperatures, this water is passed through the pads using water nozzles to cool the incoming hot air through evaporative cooling. The fan draws cold air into the poultry house, providing sufficient circulation and temperature equilibration.

### 3.2. Usable components

In Table 2, the usable component for the system has been listed. MQ 137 [22] is used to detect the toxic ammonia gases. Dust sensor [41] used to detect airborne, smoke, and pollen. Relay module [42] is used to switch voltage. BME680 [43] used to detect gas, humidity, pressure, and temperature. ESP 32 [44] takes data from other sensors. A Water flow sensor [45] monitors the water control level. BH1750 [46] to measure the surroundings of light. Capacitive soil moisture sensor [47] measure soil moisture. MQ4 [48] is used to detect methane.

**Table 2:** Component list with features and functionality.

Name	Key features and functionality
Dust Sensor	It is used to detect the airborne particles like dust, smoke and pollen.
MQ4	Used for measuring the methane concentration of the air.
MQ137	Monitoring air quality by detecting harmful gases like CO_2_, ammonia (NH_3_) etc.
Capacitive Soil Sensor	The capacitive soil sensor is used to measure soil moisture level to enhance irrigation system
BH1750	Digital Light Sensor (BH1750) uses to measure ambient light.
Water Flow Sensor	Automatically monitoring and controlling the tank’s water levels.
BME 680	It can detect gas, pressure, humidity, and temperature.
Relay Module	Relay modules are used to move switches with low power voltage to conduct high voltage.
Dust Sensor	It is used to detect the airborne particles like dust, smoke and pollen.
ESP-32	It takes data from another sensor and convert it analog to digital format.

The safety ranges, maximum allowable limits, and potential effects of various gases and environmental parameters in a poultry farming system is described in Table 3.

**Table 3:** Safety range and monitoring process of different components.

Gas/ Environmental Parameter	Recommended Range	Maximum Allowable Limit	Major Effect When Exceeded	Monitoring Method	Ref.
Ammonia	7 ppm	50 ppm	It can cause pulmonary problems and reduce egg production and poultry growth.	Gas detector, Colorimetric tubes.	[49]
Dust	2.5 mg/m^3^	15 mg/m^3^	It causes respiratory diseases for both poultry and poultry farm workers.	Gravimetric sample, real time dust monitor.	[50]
Incubation Temperature	37.5-38.0 °C	38.8-39.0 °C	It may cause poor embryo development and reduce hatchability.	Digital thermometer, incubator sensor.	[51]
Growing Temperature	21-33 °C	29 °C	It may lead to heat stress, reduced feed intake, and poor growth.	Thermometer, climate control system.	[52]
Incubation Humidity	50-60% RH	70% RH	It causes improper moisture loss and poor chick quality.	Hygrometer, incubator humidity sensor.	[53]
Hatching Humidity	65-70% RH	70% RH	It may cause sticky chicks and poor hatch rates.	Gas detector, infrared sensor.	[54]
CH4	<10 ppm	100 ppm	High levels cause asphyxiation, the greenhouse effect, and poor air quality.	Gas detector Infrared sensor.	[55]
Light	10-20 lx	60 lux	Low or high light levels negatively impact growth and welfare.	Lux meter, Automated lighting control.	[56]

### 3.3. Flowchart of IoT based smart poultry farm monitoring system

**Fig 2:**
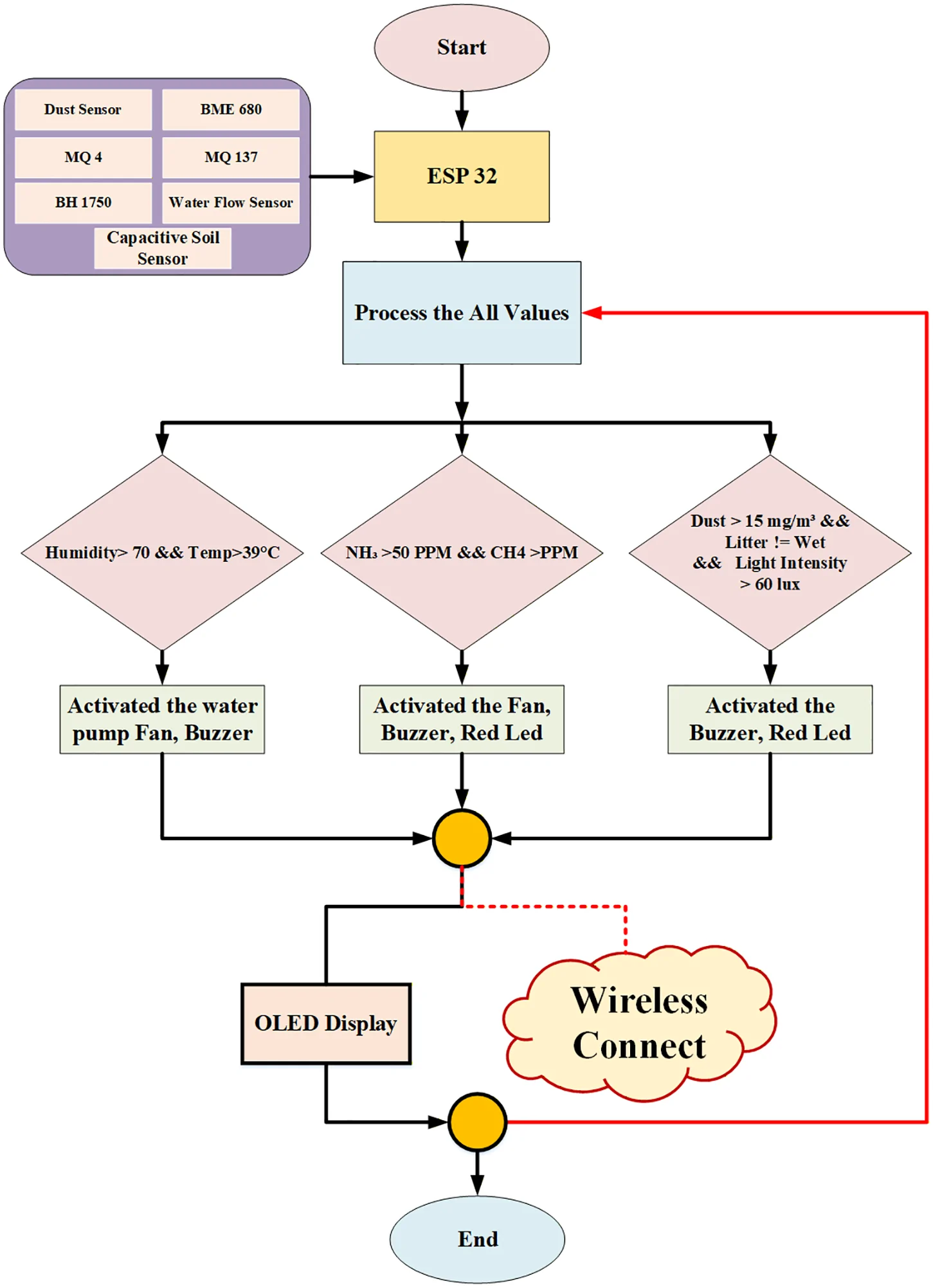
Flowchart of IoT based smart poultry farm monitoring.

In Fig 2, the flowchart illustrates how Internet of Things (IoT**)** based Smart Poultry Farm Monitoring works with the ESP32 microcontroller. According to the schematic architecture, environmental data are first collected by sensors, including dust, gas (MQ4 and MQ137), temperature & humidity (BME 680), light (BH1750), water flow, and capacitive soil. All sensor values are processed by the ESP32, which ultimately triggers decisions based on certain thresholds for communication. If humidity > 70% and temperature > 39°C, the system turns on the water pump, fan, and buzzer. If NH_3_ (>50 PPM) or CH_4_ (10 ppm ≤ CH_4_ ≤ 100 ppm), turn on the fan, buzzer, and Red LED. When the dust concentration is 15 mg/m³, the litter is wet, and the light intensity is too strong to ring the buzzer or activate the red LED. This information is also available remotely through a connected device. This process continues as the exchange is recycled, making it a feedback loop that helps achieve an ideal poultry environment**.**

### 3.4. Data management of IoT based smart poultry farm system

**Fig 3:**
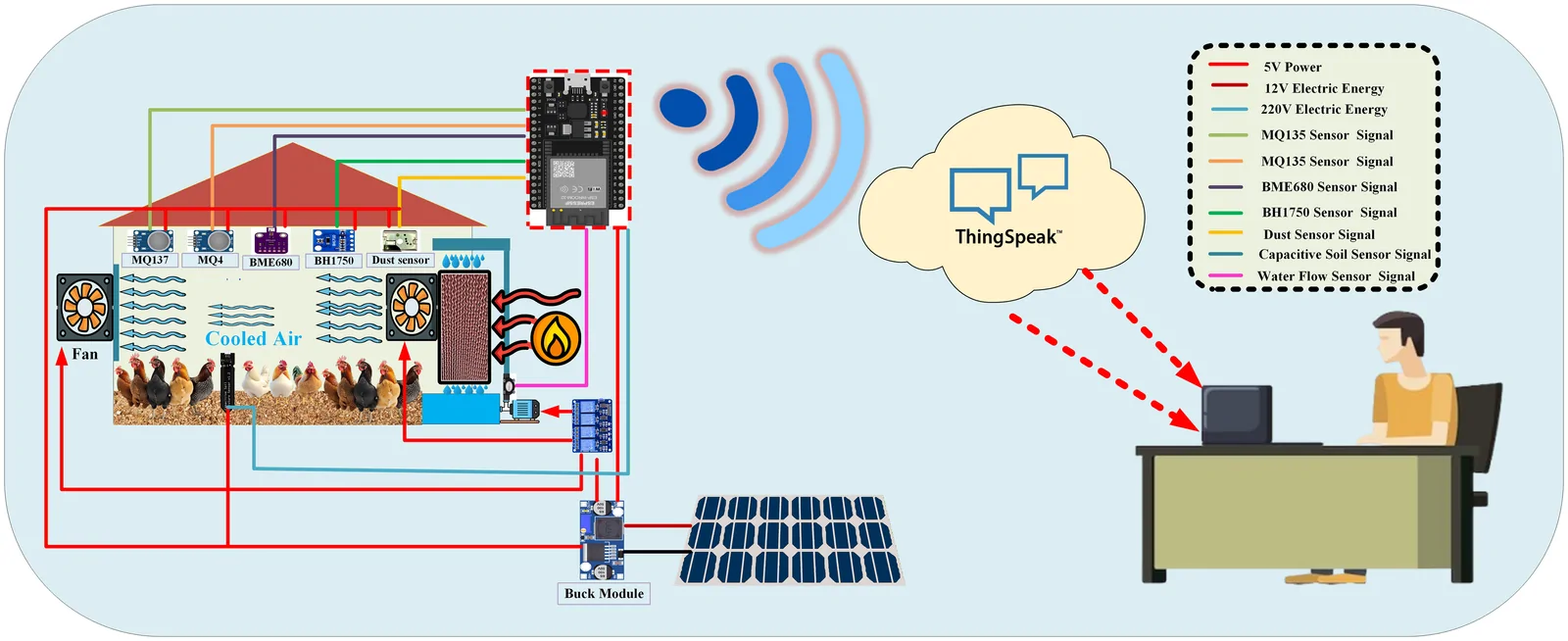
Poultry farm system architecture with remote monitoring.

Fig 3 is the structure of a smart poultry farm, indicates how it handle all the collected data. This Internet of Things (IoT) based system continuously monitor the farm environment using various sensors. A microcontroller collects the data from the sensor. Based on real time data, the system controls the fan and cooling system to maintain a healthy environment inside the farm. The real-time data is also sent to the cloud platform wirelessly, which helps the farmer to monitor the farm environment and take necessary steps. The system also incorporates solar panels to power the entire setup, ensuring sustainability and reducing reliance on external electricity sources. The processed data is wirelessly transmitted to ThingSpeak, a cloud platform, which allows real-time monitoring and management of the system from a remote computer.

### 3.5. Schematic diagram

**Fig 4:**
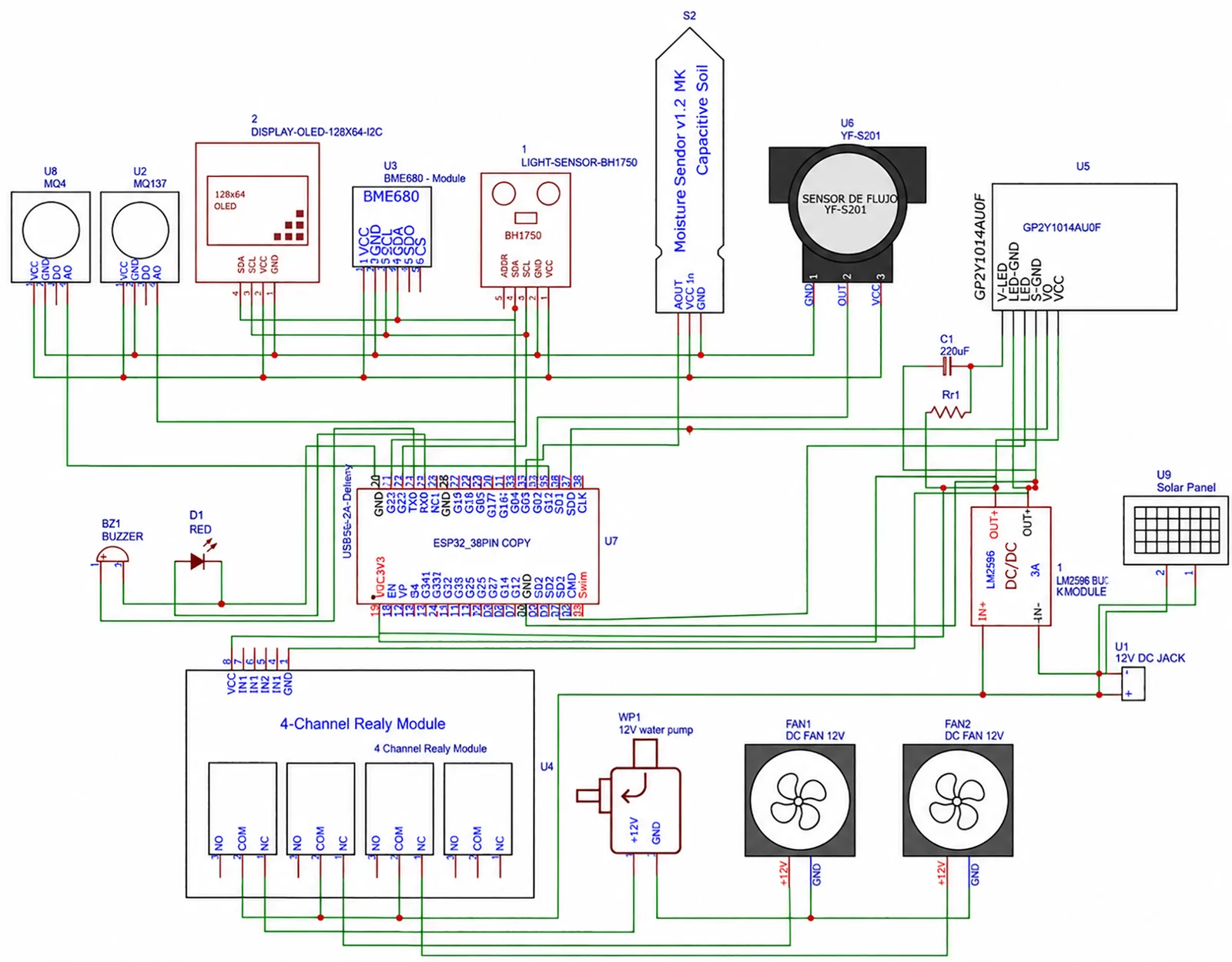
Schematic diagram of the system.

The circuit diagram of an Internet of Things (IoT) based smart poultry farming system is depicted in Fig 4. The ESP32 acts as the central microcontroller, processes data from the sensors, and controls components such as the water pump, DC fans, and other devices via a four-channel relay module. The system also has an OLED display for data viewing in real time, and a solar panel to power the system indefinitely. The figure also illustrates how the modules are powered, interconnected and function together to keep the poultry farm in a desirable state.

### 3.6. Pseudocode of the proposed system

**Algorithm 1: IoT-based automated early detection and environmental monitoring for poultry farming**

// Input Variables:

1: T_s ← Temperature sensor data (BME680, °C)

2: H_s ← Humidity sensor data (BME680, % RH)

3: NH3_s ← Ammonia sensor data (MQ137, PPM)

4: CH4_s ← Methane sensor data (MQ4, PPM)

5: D_s ← Dust sensor data (mg/m³)

6: L_s ← Light intensity sensor data (BH1750, lux)

7: SM_s ← Soil moisture sensor data (Capacitive sensor, %)

8: WF_s ← Water flow sensor data (L/min)

// Output Variables:

9: F_ctrl ← Fan/ventilation control status (ON/OFF)

10: P_ctrl ← Water pump control status (ON/OFF)

11: A_alert ← Audio buzzer alert status (ON/OFF)

12: L_alert ← LED alert status (RED/GREEN)

13: D_cloud ← Data transmission to ThingSpeak cloud

//Threshold Constants:

14: T_max = 39°C

15: H_max = 70% RH

16: NH3_max = 50 PPM

17: CH4_max = 10 ppm =<CH4 <= 100 ppm

18: D_max = 15 mg/m³

19: L_max = 60 lux

// Initialize System:

20: Initialize ESP32 microcontroller

21: Initialize all sensors: BME680, MQ137, MQ4, BH1750, Dust, Capacitive soil, Water flow

22: Initialize relay module, buzzer, LED indicators, OLED display

23: Establish Wi-Fi connection to ThingSpeak cloud platform

24: While true:

  // Step 1: Read all sensor data

25:    T_s = ReadSensor(BME680_Temperature)

26:    H_s = ReadSensor(BME680_Humidity)

27:    NH3_s = ReadSensor(MQ137_Ammonia)

28:    CH4_s = ReadSensor(MQ4_Methane)

29:    D_s = ReadSensor(DustSensor)

30:    L_s = ReadSensor(BH1750_Light)

31:    SM_s = ReadSensor(CapacitiveSoilMoisture)

32:    WF_s = ReadSensor(WaterFlowSensor)

  // Step 2: Display data on OLED

33:     Display (T_s, H_s, NH3_s, CH4_s, D_s, L_s, SM_s, WF_s) on OLED

  // Step 3: Check temperature and humidity thresholds

34:    If (T_s > T_max) OR (H_s > H_max):

35:    F_ctrl = ON

36:    P_ctrl = ON

37:    A_alert = ON

38:    L_alert = RED

39:    Else:

40:    F_ctrl = OFF

41:    P_ctrl = OFF

  // Step 4: Check gas concentration thresholds

42:    If (NH3_s > NH3_max) OR (CH4_s > CH4_max):

43:    F_ctrl = ON// Activate fan for gas removal

44:    A_alert = ON

45:    L_alert = RED

46:    Else:

47:    L_alert = GREEN

  // Step 5: Check dust and light thresholds

48:    If (D_s > D_max) OR (L_s > L_max):

49:    A_alert = ON// Trigger buzzer alert

50:    L_alert = RED

  // Step 6: Transmit data to ThingSpeak cloud

51:    If (Wi-Fi connected):

52:    D_cloud = Transmit (T_s, H_s, NH3_s, CH4_s, D_s, L_s, SM_s, WF_s)

53:    to ThingSpeak via HTTP protocol

54:    Else:

55:    Attempt reconnection to Wi-Fi

56:    Wait (30 seconds)

57: End While

## 4. Sensor validation

Table 4 compares the sensors used to detect temperature, humidity, ammonia, methane, lux, and dust, assessing their accuracy and reliability in an Internet of Things (IoT**)** based system for monitoring poultry houses. A total of 15 days of data were collected for the study.

**Table 4:** Recorded sensor data for environmental monitoring in poultry farming.

Sl No	UT333s Temperature (°C)	BME680 Temperature (°C)	UT333s Humidity (%)	BME680 Humidity (%)	BT-5800G Ammonia (PPM)	MQ137 Ammonia (PPM)	CGD02A Methane (PPM)	MQ4 Methane (PPM)	UNI-T UT383 (lux)	BH1750 (lux)	Aerosol Monitor 8530 Dust (mg/m^3^)	Dust Sensor (mg/m^3^)
1	30	31	67	66	0	0	3	2	73	74	3	3
2	32	33	70	69	2	1	7	6	12	12	6	5
3	27	29	72	71	4	4	1	0	60	60	0	0
4	25	26	74	73	3	2	0	0	30	31	0	1
5	30	29	65	63	7	8	2	1	82	82	1	1
6	34	33	69	68	9	9	2	3	48	48	2	3
7	29	30	63	65	5	4	6	5	13	12	4	5
8	30	32	71	72	3	3	6	7	98	98	1	2
9	34	32	70	70	2	1	10	9	23	23	3	4
10	29	30	65	66	10	9	0	0	18	18	2	2
11	35	34	68	68	8	7	3	3	62	62	3	2
12	29	29	69	70	6	7	4	4	60	61	0	0
13	35	36	65	63	12	11	8	7	27	27	1	2
14	30	30	61	60	9	10	2	1	48	47	3	2
15	32	31	60	58	4	6	0	0	69	69	2	1

**Fig 5:**
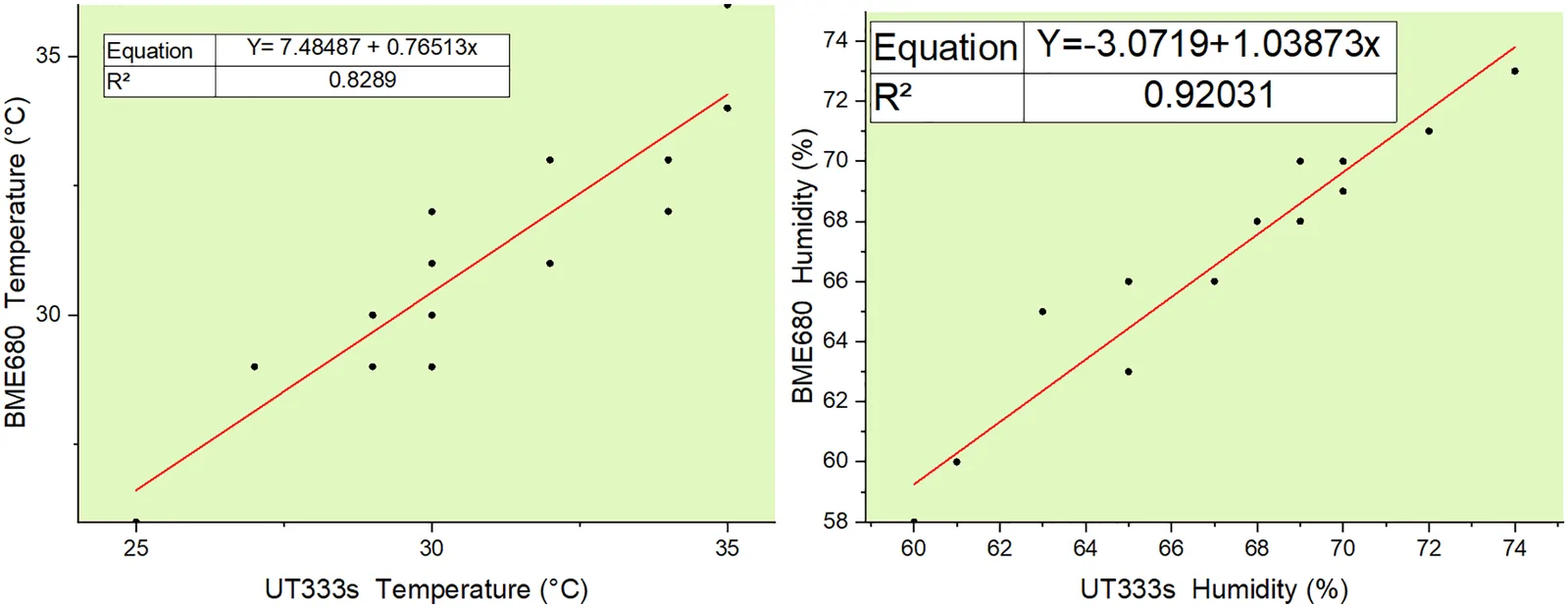
Linear co-relation of BME-680 with UT333s as temperature and humidity.

**Fig 6:**
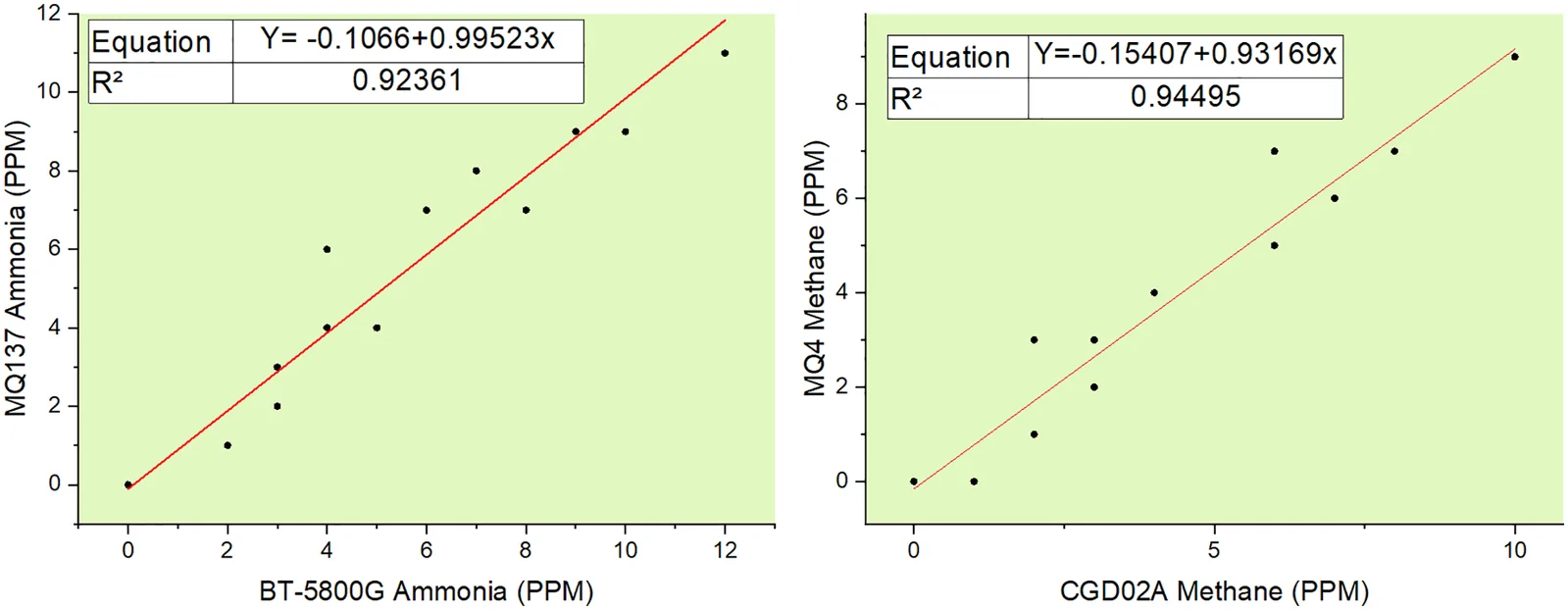
Linear correlation between ammonia and methane sensor readings.

**Fig 7:**
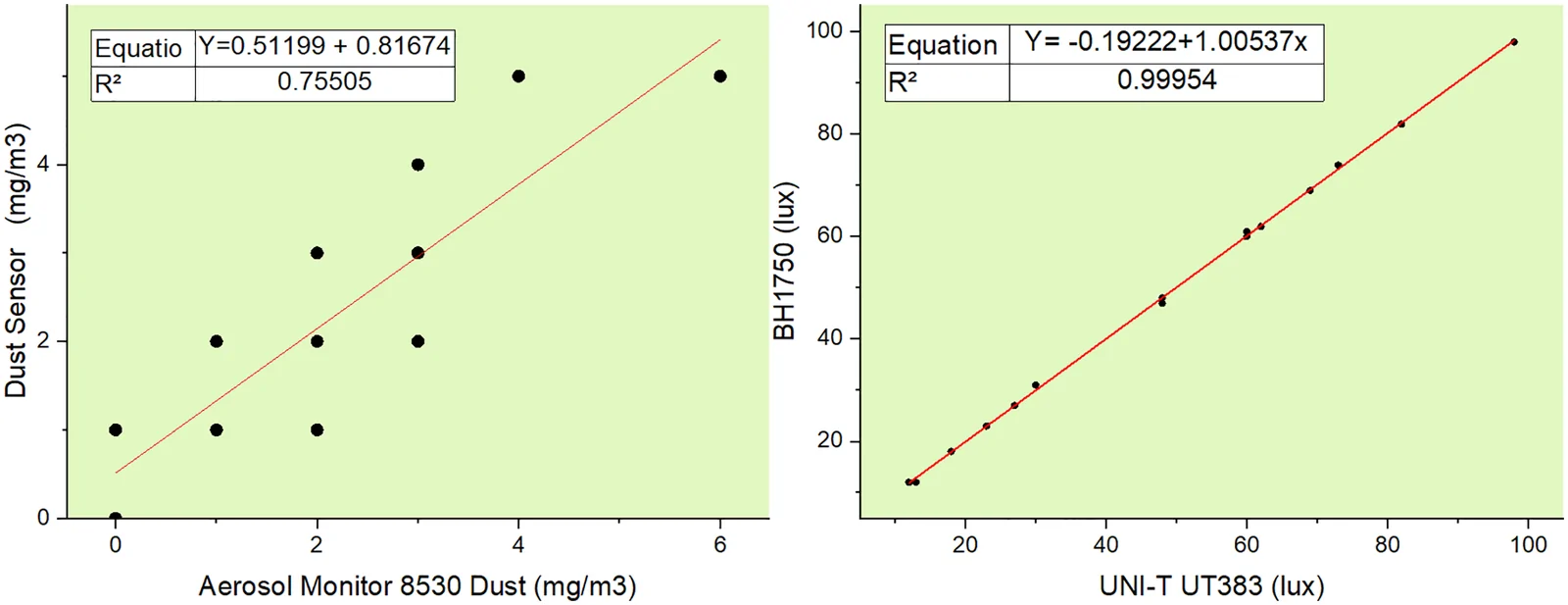
Correlation between dust sensor and light sensor readings.

To better understand the prototype model sensors, a compressive linear correlation method was used with commercially available devices. In Figs 5 - 7, the correlation between the prototype model sensor and a commercially available sensor is plotted. A significant correlation between BME680 and UT333s data is evident in Fig 5, with humidity readings showing slightly higher agreement (a higher R^2^) than temperature readings.

Both sensors (MQ137 and MQ4) correlate strongly with their respective reference sensors depicted in Fig 6. The methane sensor shows a slightly stronger correlation (a higher R^2^). Small negative intercepts in both equations suggest a slight underestimation at low concentrations.

Dust sensor correlates reasonably well with the aerosol monitor (R^2^ = 0.75505). Light sensors (BH1750 and UNI-T UT383) show excellent agreement (R^2^ = 0.99954), suggesting near identical performance. Graphical plotting of these two sensors is depicted in Fig 7.

### 4.1. Descriptive analysis of data from commercial devices and sensors

In Table 5, comparison and analysis of data collected from various commercially available sensors and the sensor integrated into the prototype are depicted. The purpose of the comparison is to show how accurately the prototype sensor performs compared to standard market devices under similar environmental conditions. For better comparison, 15 days of data have been surveyed for both the prototype sensor and commercially available sensors. During the comparison, the results were very similar, and in some cases, the prototype sensor performed better. For temperature measurement, both the BME680 and UT333S commercial sensors provide similar results and exhibit very low error margins. A similar result was found for humidity measurement. For ammonia concentration, both the commercial sensor and the sensor in our prototype provide a mean value of 5–6 ppm. Also, the error difference was very small, indicating that the prototype can detect harmful gases with high accuracy. For methane detection, both the prototype and commercial sensors provide values around 5–6 ppm. Overall, the comparison indicates that the prototype developed in this study achieves performance comparable to that of standard market sensors.

**Table 5:** Sensor data summary with statistical analysis.

Device	UT333s Temperature (°C)	BME680 Temperature (°C)	UT333s Humidity (%)	BME680 Humidity (%)	BT-5800G Ammonia (PPM)	MQ137 Ammonia (PPM)	CGD02A Methane (PPM)	MQ4 Methane (PPM)	UNI-T UT383 (lux)	BH1750 (lux)	Aerosol Monitor 8530 Dust (mg/m^3^)	Dust Sensor (mg/m^3^)
Sample Size	15	15	15	15	15	15	15	15	15	15	15	15
Minimum	25	26	60	58	0	0	0	0	12	12	0	0
Maximum	35	36	74	73	12	11	10	9	93	98	8	7
Mean	30.73	31	67.26	66.80	5.60	5.20	4.53	3.33	48.06	48.46	2.86	2.60
Std. Error of Mean	0.75	0.63	1.04	1.13	0.89	0.90	0.86	0.74	6.63	6.82	0.56	0.53
Standard Deviation	2.91	2.44	4.04	4.37	3.45	3.48	3.33	2.89	25.69	26.44	2.19	2.06
Variance	8.49	6.00	16.35	19.17	11.97	12.17	11.12	8.38	660.20	699.40	4.83	4.25

### 4.2. Average daily assessment

**Fig 8:**
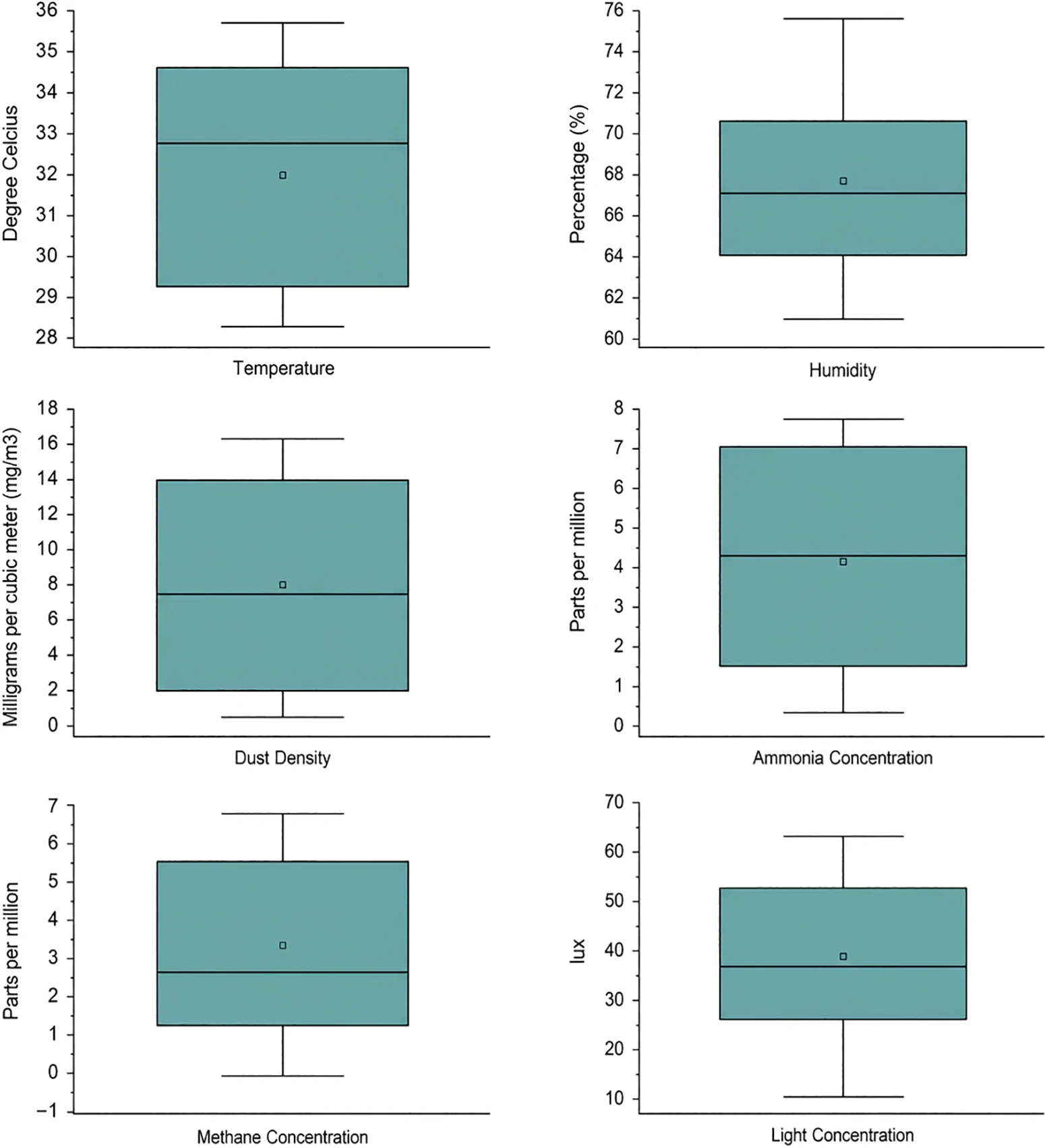
Box plots of environmental parameters in poultry farm.

In Fig 8, over 15 days in August, the conditions inside the poultry farms: temperature, humidity, dust density, ammonia, methane, and light concentration were monitored. These details were recorded every half hour, gathering 48 measurements daily, for a total of 720 per month. The data charts were analyzed, showing daily averages as bar heights, total values as stacked columns, and separate lines indicating high and low ranges, making it easier to interpret fluctuations and trends. This research aimed to determine whether the surrounding environment impacted poultry farming.

### 4.3. Environmental parameter analysis for poultry farm over 15 days

The environmental parameters were analyzed, and the results are presented in Table 6. For each parameter, the table provides mean values, SD, SEM, and the number of times the safe limits were exceeded (Unsafe count). The numerical data for quality (dust concentration, ammonia concentration, methane concentration, and light concentration) are presented daily over a month. This table presents the variations in interior temperature, relative humidity, dust concentration, ammonia concentration, methane concentration, and light intensity with the day and date over a 15-day period of daylight in August. The relatively low SEM and SD findings for this study are promising.

**Table 6:** Daily environmental data and sensor readings for poultry farm.

Day	Temperature Mean (°C)	Temperature SEM	Temperature SD	Unsafe Count Temperature	Humidity Mean (%)	Humidity SEM	Humidity SD	Unsafe Count Humidity	Dust Concentration Mean (mg/m3)	Dust Concentration SEM	Dust Concentration SD	Unsafe Count Dust Concentration	Ammonia Concentration Mean (PPM)	Ammonia Concentration SEM	Ammonia Concentration SD
Day 1	30.99	0.20	1.01	3	63.66	0.87	1.69	20	10.32	0.82	1.32	1	5.30	0.22	0.24
Day 2	35.60	0.74	0.67	7	66.08	1.11	2.45	1	2.89	0.90	1.53	0	2.49	0.30	0.68
Day 3	33.85	0.78	1.88	14	70.49	1.63	2.79	2	1.10	0.38	1.47	1	4.16	0.381	0.33
Day 4	32.78	0.60	1.81	20	68.63	0.84	2.77	4	16.13	0.19	0.83	2	4.37	0.24	0.63
Day 5	29.24	0.79	0.88	13	65.82	0.61	2.55	19	16.41	0.30	1.56	1	1.47	0.48	0.47
Day 6	29.24	0.54	1.48	9	72.23	0.93	2.28	1	13.74	0.48	0.85	3	7.75	0.48	0.57
Day 7	28.46	0.57	1.72	0	62.78	0.74	1.168	0	5.17	0.83	0.98	0	6.20	0.20	0.40
Day 8	34.92	0.48	1.33	12	65.84	1.89	1.32	11	1.66	0.87	1.61	0	7.51	0.29	0.44
Day 9	32.80	0.12	1.29	4	67.32	1.71	2.79	1	11.63	0.10	1.47	0	7.15	0.22	0.39
Day 10	33.66	0.19	0.86	5	69.12	1.45	2.21	3	7.48	0.55	1.77	1	4.78	0.21	0.21
Day 11	28.16	0.12	0.63	6	75.70	1.80	1.01	5	2.07	0.47	1.48	1	7.37	0.11	0.53
Day 12	35.75	0.67	1.84	19	63.99	1.70	1.20	12	8.41	0.29	1.35	1	0.70	0.34	0.26
Day 13	34.65	0.38	1.85	4	70.28	0.77	2.32	5	0.58	0.20	0.64	1	1.56	0.30	0.11
Day 14	29.69	0.55	1.44	1	71.84	1.83	1.01	1	15.45	0.40	1.05	0	0.36	0.12	0.48
Day 15	29.45	0.91	1.00	3	60.92	1.30	1.32	0	4.39	0.94	0.89	0	2.60	0.21	0.20

**Fig 9:**
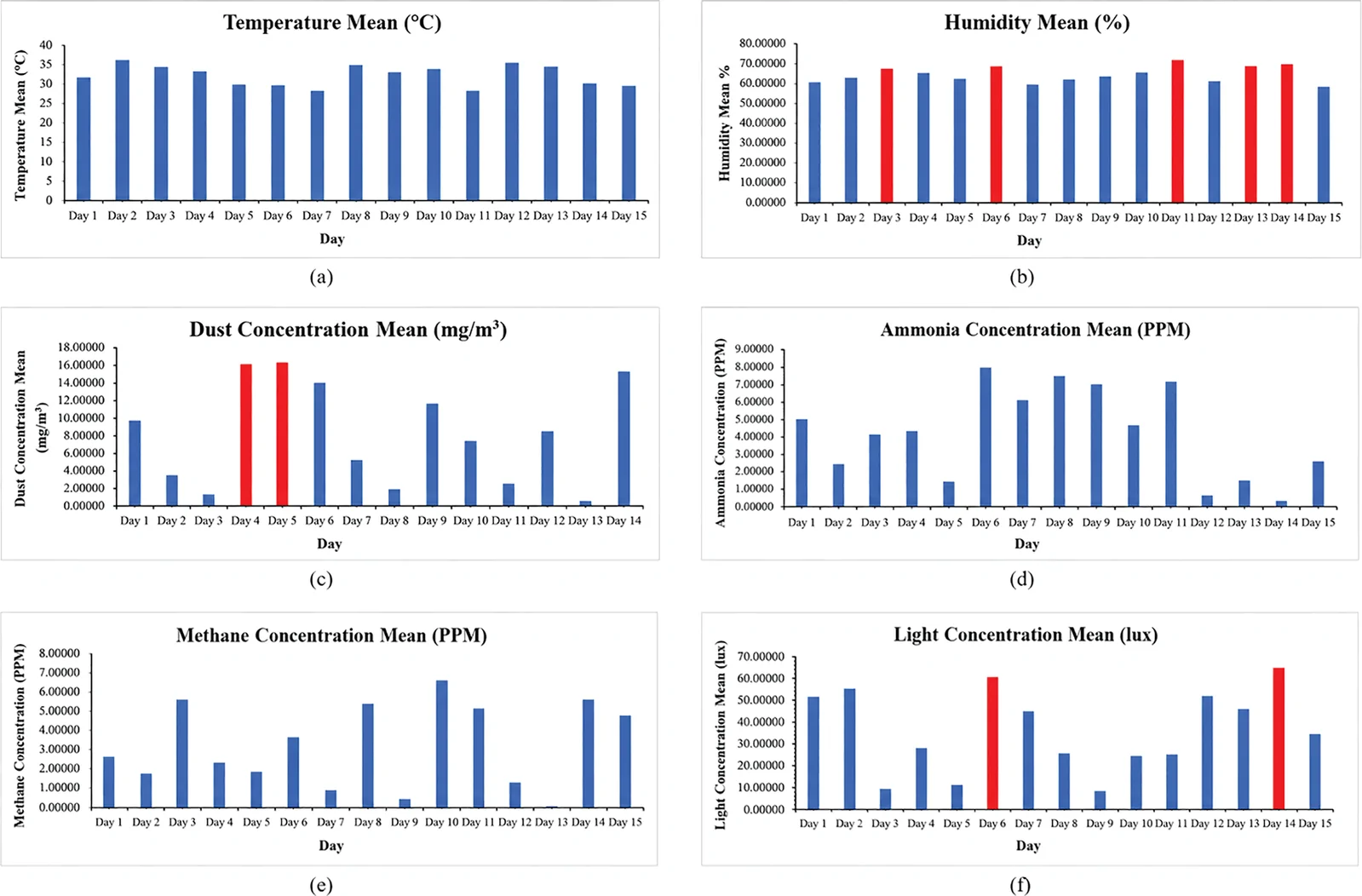
Environmental factors in a poultry farm over 15 days temperature, humidity, dust, ammonia, methane, and light concentrations.

Fig 9 shows six bar graphs of environmental factors in a poultry farm over 15 days. Temperature fluctuates from 30°C on Day 1–35°C on Day 2, dropping to 27–29°C by Day 7, then increasing again to 34°C before stabilizing at 28–29°C on Days 14–15 depicted in Fig 9 (a). In Fig 9 (b) Humidity starts at 60–65%, rising to 70% on Days 3–6, then stabilizing at 60–65% with spikes on Days 11–14, returning to 60% on Day 15. In Fig 9 (c) dust concentration drops from 10 mg/m³ on Day 1–2–3 mg/m³ on Days 2–3, rising again on Days 4–5, then decreasing toward the end. Ammonia concentration starts at 5 PPM, drops to 1 PPM on Day 5, and rises again on Days 6–9 before stabilizing towards the end depicted in Fig 9 (d). In Fig 9 (e) methane concentration rises to 6 PPM on Day 3, peaks at 7–8 PPM on Days 10–11, then decreases to 5 PPM by Day 15. Finally in Fig 9 (f) Light concentration fluctuates from 50–55 lux on Days 1–2, drops on Day 3, and rises again on Days 6, 14, with stability at 45–50 lux on other days.

Table 7 presents a 15 days analysis of temperature, humidity, dust concentration, ammonia concentration, methane concentration, and light concentration in a poultry house throughout August. The sample size for each parameter was 15, which corresponds to August’s 15 days, with mean values of 32.71°C for temperature, 67.23% for relative humidity, 5.33 µg/cm³ for dust concentration, 4.89 PPM for ammonia concentration, 4.23 PPM for methane concentration, and 50.7 for light concentration. The data showed that 20% dust concentration, 33.33% humidity, and 13.33% light concentration readings were beyond the permissible range; however, none of the temperature, ammonia concentration, or methane concentration readings were above the threshold.

**Table 7:** Environmental parameters and their statistical analysis in poultry house.

Parameters	Temperature (°C)	Humidity (%)	Dust Concentration (mg/m³)	Ammonia Concentration (PPM)	Methane Concentration (PPM)	Light Concentration (lux)
**Sample Size**	15	15	15	15	15	15
**Mean**	32.71	67.23	5.33	4.89	4.23	50.7
**Std. Error of Mean**	0.27	0.61	1.24	0.23	0.1	1.8
**Median**	33.86	66.08	3.43	4.22	3.97	51.15
**Mode**	33.86	66.08	3.42	4.22	3.97	51.15
**Range**	7.48	6.9	15.26	3.42	3.96	19.42
**Minimum**	28.16	60.93	1.17	2.49	1.9	36.75
**Maximum**	35.61	76.64	16.42	5.73	5.92	58.31
**Std. Deviation**	1.10	2.29	5.46	0.87	0.39	5
**Maximum Allowable Limit**	39.0°C	70% RH	15 mg/m³	50 ppm	100 ppm	60 lx
**Outside Safe Range %**	0%	33.33%	20%	0%	0%	13.33%
**No. of Values Outside Safe Range**	0	5	3	0	0	2

In Table 8, environmental conditions were checked against reference levels. The temperature reached 31.96°C; humidity was 67.65%, and dust measured 7.83 mg/m^3^. Ammonia: 4.26 ppm; methane: 3.35 ppm; illumination: 37.63 lux. Finally, after all the evaluations, it is observable that everything stayed below established safety thresholds.

**Table 8:** Monthly average and maximum limits of environmental parameters in the poultry house.

Parameter	Monthly mean of 15 days	Maximum limit	Comment
**Temperature**	31.96°C	39.0°C	Within safe range
**Humidity**	67.65%	70.00%	Within safe range
**Dust Density**	7.83 mg/m³	15.0 mg/m³	Within safe range
**Ammonia Concentration**	4.26 ppm	50.0 ppm	Within safe range
**Methane Concentration**	3.35 ppm	100.0 ppm	Within safe range
**Light Concentration**	37.63 lux	60.0 lux	Within safe range

## 5. Prototype preparation and validation with the existing model

**Fig 10:**
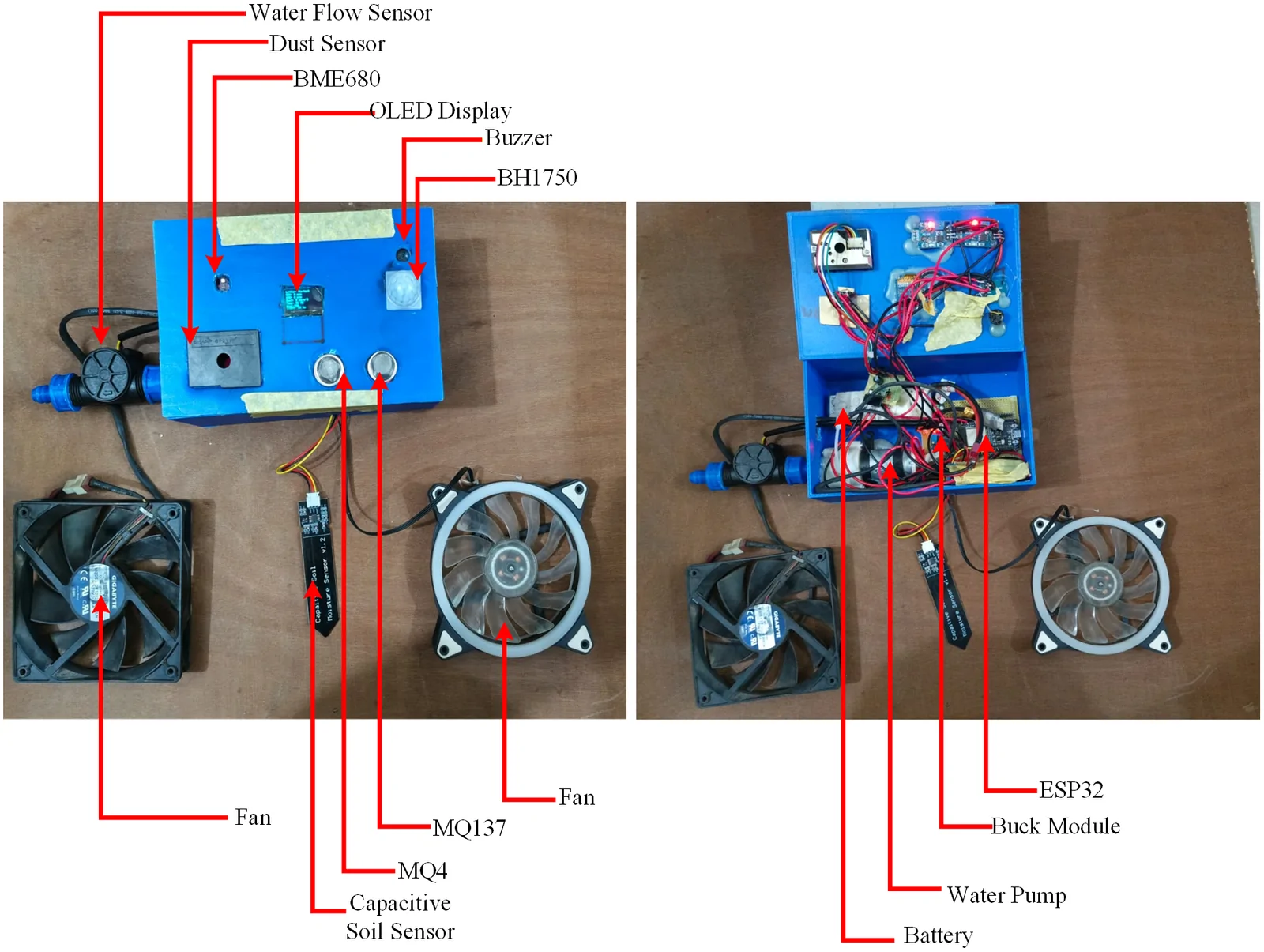
Prototype model with component labels.

The prototype model developed in [17,18] is not strong enough to provide the actual data. The 3D structure is not strong enough, and the device’s durability is also poor, whereas this study prototype has undergone extensive testing. In Fig 10, the prototype model is depicted. Several parts were indicated by an arrow sign. The box is 3D printed, strong, and durable. The system has a control unit enclosed in a blue casing and is connected to two cooling fans placed on a stable surface. The left side picture shows the internal circuit board, where a microcontroller, a breadboard, and other electronic components are connected. These components receive sensor data to control fan operation. The system is powered through external wiring to ensure uninterrupted operation. The view from the right-side picture shows that the control unit is operating and the cover is closed. At the top, there is a digital display, push buttons, and a sensor installed, which allows real time temperature monitoring and manual control. The cooling fans are connected to the control box, and the microcontroller controls their speed based on sensor inputs.

**Fig 11:**
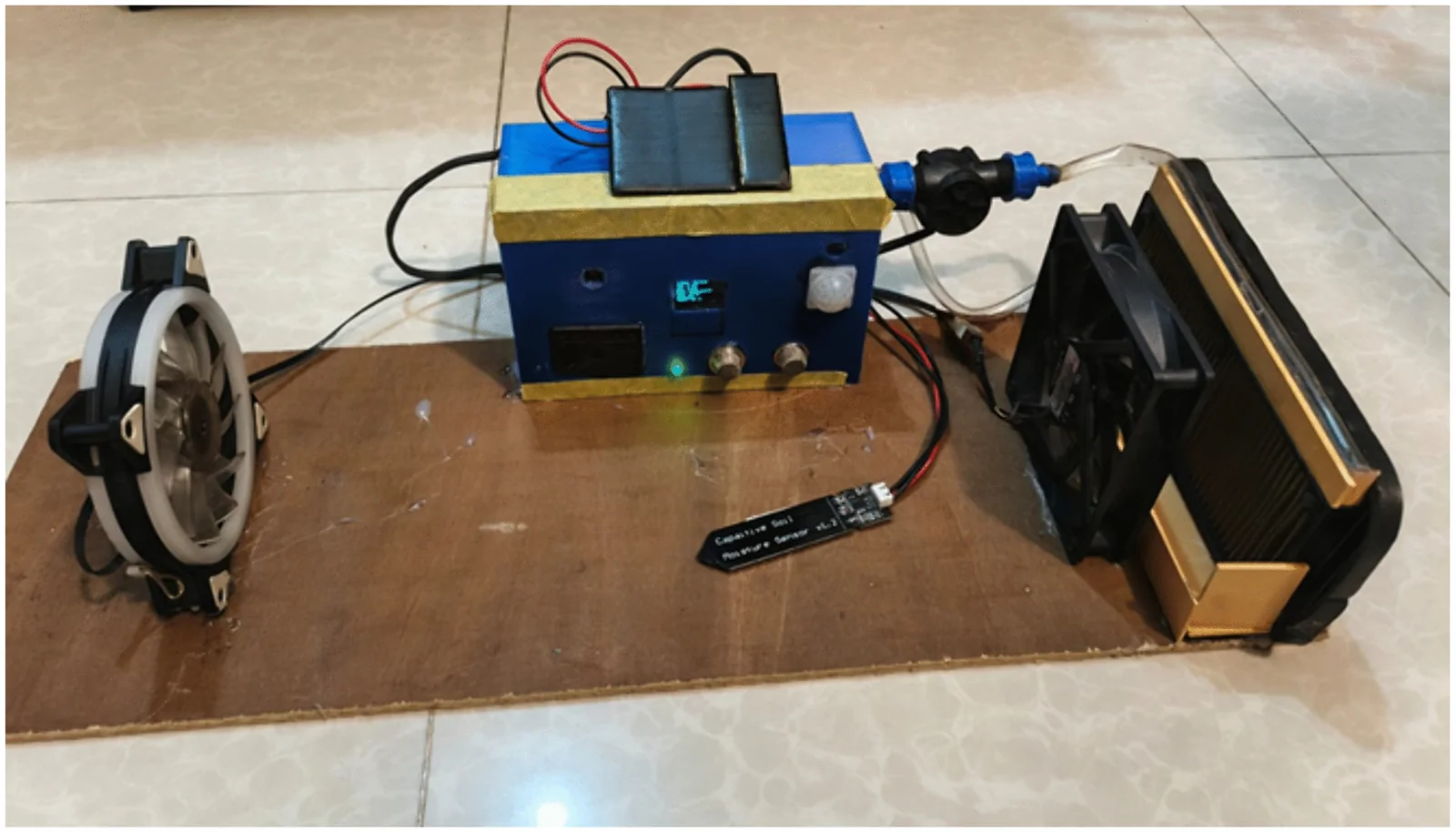
Solar powered automated poultry house monitoring system.

A complete setup of the prototype model with a solar panel is depicted in the Fig 11. It contains a water pump inside and a water flow sensor on top. It has two fans, one attached to the honeycomb pad and the other used to release hot air outside. The system uses a water flow sensor to circulate water through a honeycomb pad, which helps cool the air, and then the fan attached to the honeycomb pad blows the cooled air into the surrounding area.

In Table 9 represents a comparison between the sensors used in this proposed system and those in previous studies related to poultry farms. From the table, it can be seen that the proposed system includes all the major environmental parameters relevant to poultry farming, such as temperature, humidity, dust density, gas concentrations, luminosity, litter condition, and an early alarming system all integrated using Internet of Things (IoT) technology. In contrast, other studies have used only a few selected sensors for monitoring; for example, some previous works did not include temperature, humidity, or gas concentration sensors, while others lacked an early alarming system. Therefore, this proposed system is more comprehensive and effective in providing better environmental monitoring for poultry farms compared to previous works.

**Table 9:** Comparison between the present work and earlier research.

Reference	Farm Type	Environmental Parameters							Earlier Alarming System	Technology
		Temperature	Humidity	Dust Density	Ammonia Concentration	Methane Concentration	Luminosity	Litter Status		
[22]	Poultry houses	**✔**	**✔**	**✖**	**✔**	**✔**	**✖**	**✖**	**✔**	IoT
[57]	Poultry farm	**✔**	**✖**	**✖**	**✖**	**✖**	**✖**	**✖**	**✖**	IoT
[32]	Poultry farm	**✔**	**✔**	**✖**	**✔**	**✖**	**✔**	**✖**	**✖**	IoT
[26]	Poultry houses	**✔**	**✔**	**✖**	**✖**	**✖**	**✔**	**✖**	**✖**	IoT
[58]	Livestock farm	**✔**	**✔**	**✖**	**✖**	**✖**	**✖**	**✖**	**✖**	IoT
[59]	Livestock farm	**✔**	**✖**	**✖**	**✖**	**✖**	**✔**	**✖**	**✖**	IoT
This paper	Poultry farm	**✔**	**✔**	**✔**	**✔**	**✔**	**✔**	**✔**	**✔**	IoT

### 5.1. Prototype testing

**Fig 12:**
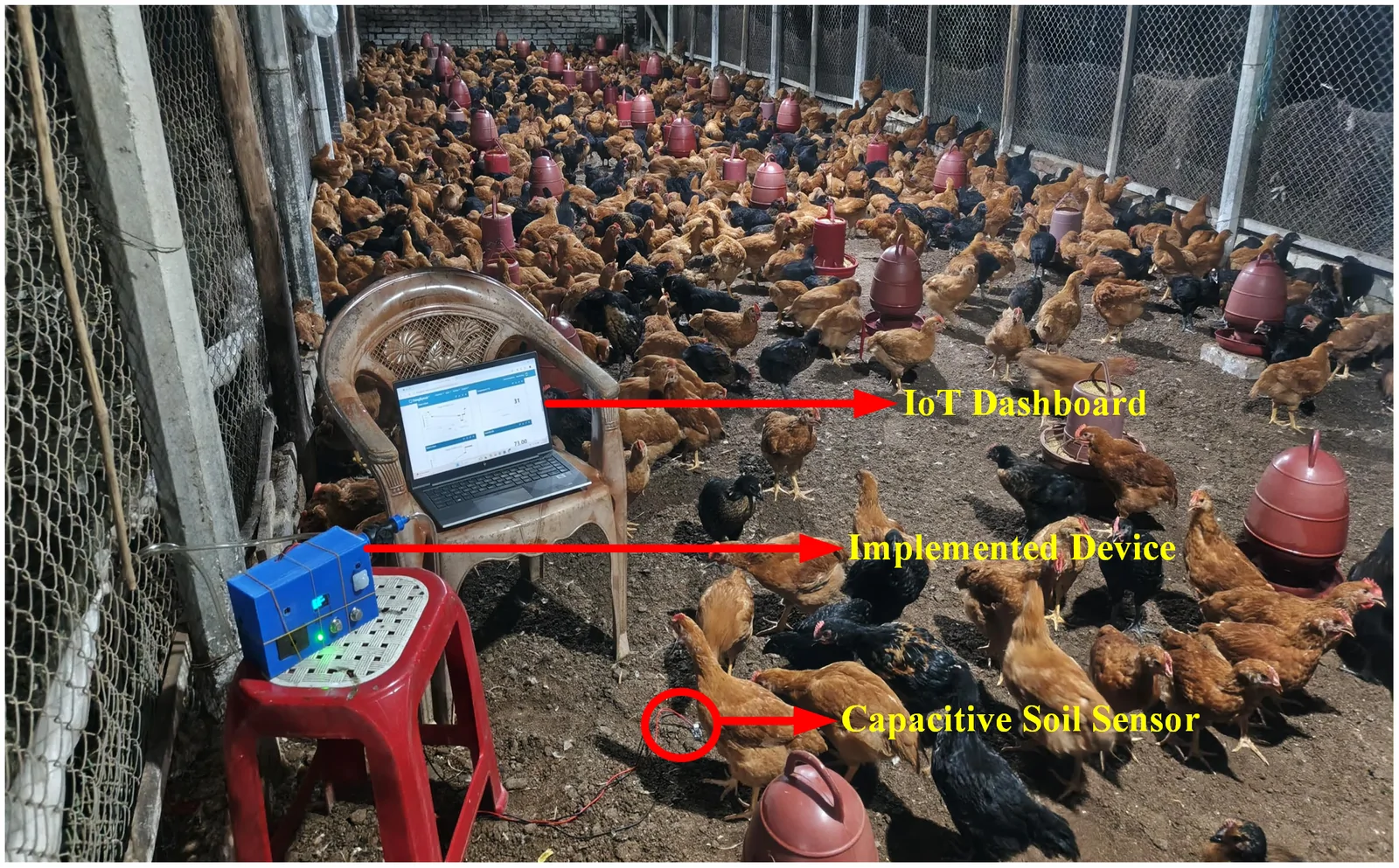
Real Time environmental data monitoring in a poultry farm using IoT devices.

In Fig 12, a prototype for a poultry farm has been developed to monitors real time environmental conditions, including dust density, concentrations of harmful gases such as methane and ammonia, light intensity, and litter status, using multiple sensors. To test the prototype under real world environmental conditions, a visit was made to a poultry farm where it was used. During testing, the prototype successfully monitored various environmental parameters at the poultry farm, including dust density, humidity, light intensity, litter condition, and concentrations of harmful gases (methane and ammonia). The acquired data is visualized on a wirelessly connected laptop through the ThingSpeak Internet of Things (IoT) base platform. This process was repeated for more than 15 days, and the collected data are also presented in Tables IV, V, and VI. To improve performance, the prototype model was moved to a different location within the farm. Fig 12 is licensed under CC BY 4.0.

## 6. Result, cost analysis & conclusion

**Fig 13:**
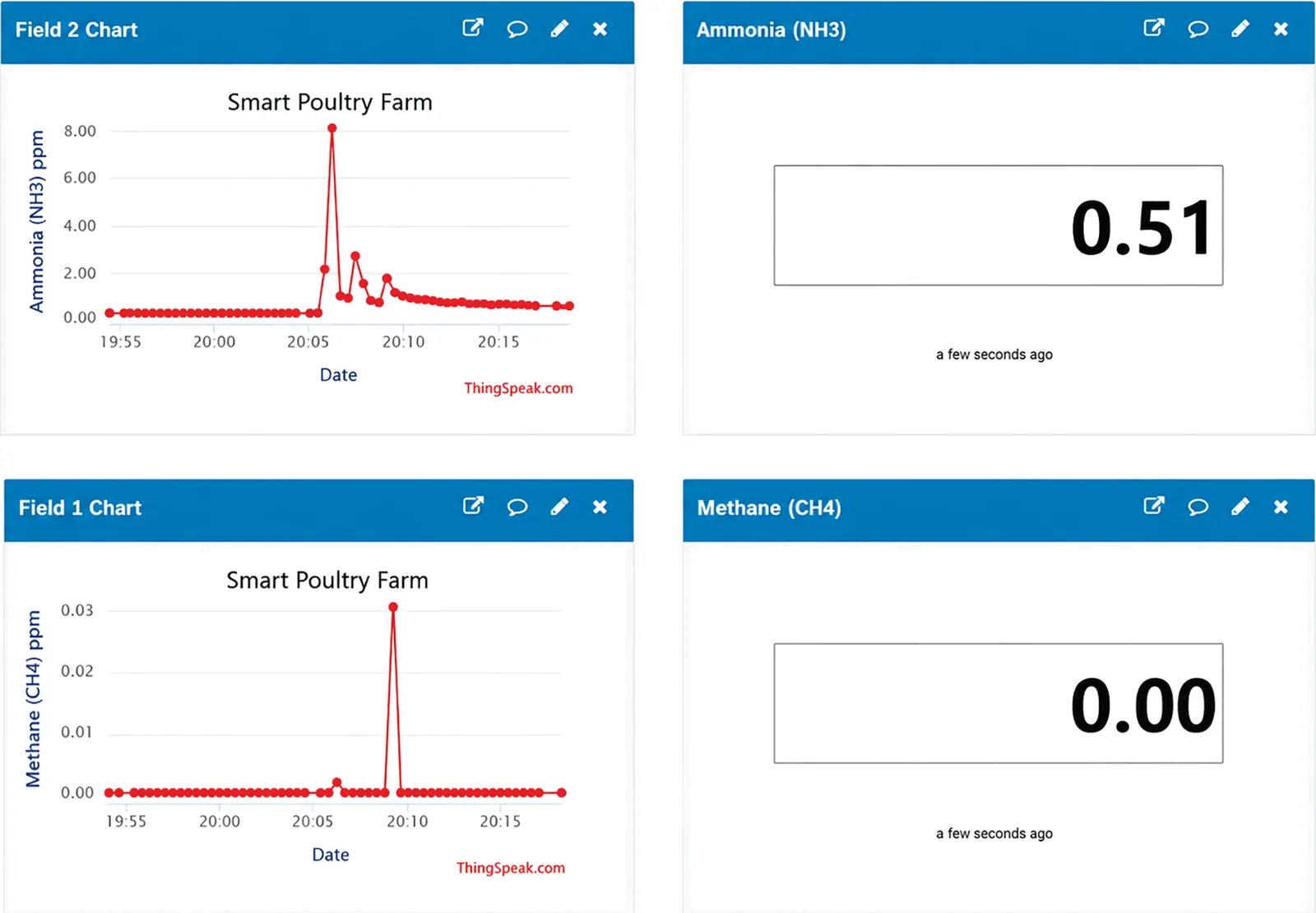
Ammonia and methane level monitoring in real time.

**Fig 14:**
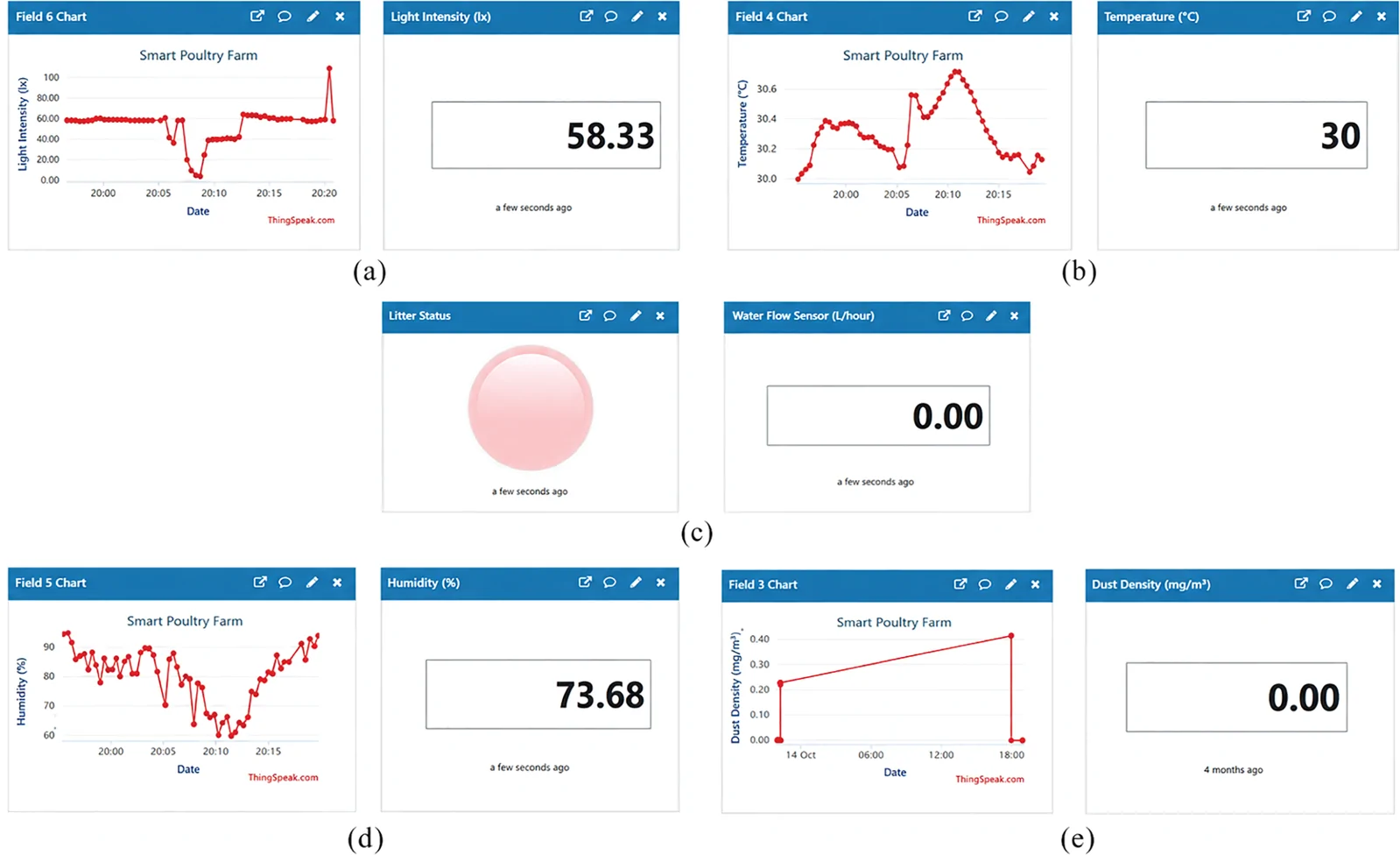
Poultry farm IoT dashboard monitoring.

The results reported in [60] are insufficient to monitor a large-scale poultry farm. Additionally, the author did not do extensive prototype testing. In our developed model, the results shown in Figs 13 and 14 are assessed in real time, and the stored 15-day data are previously depicted in Tables IV, V, and VI. In Fig 13, the upper figure represents the ammonia level, and the lower figure represents the methane level in the poultry farm. These two gases are harmful to any poultry farm, which is why real time monitoring is necessary. If, by any chance, these two gases exceed the safety level, the buzzer will activate.

In Fig 14 (a), the variation in light intensity from the digital light sensor BH1750 is shown. Similarly, Fig 14 (b) shows the real time temperature of the poultry farm, and Fig 14 (c) shows the litter status, which turns red when the litter is wet. On the other hand, when the temperature rises, the water flow will be automatically activated to reduce the farm’s excessive temperature. Fig 14 (d) and (e) represents the real time humidity and dust level of the poultry farm.

In Table 10, this study compares the proposed system with several state-of-the-art IoT based poultry monitoring solutions from the literature in terms of monitored parameters and implementation aspects. Previous systems usually monitor two to four parameters and rarely undergo longitudinal field validation, whereas our device integrates seven environmental sensors that have been continuously assessed for 15 consecutive days in a commercial poultry farm, with measurements being validated against market available reference sensors. The proposed platform is especially suitable to resource limited farms; unlike systems based solely to basic alarms or GSM messaging, it combines early warning alerts, automated evaporative cooling, solar power supply, and ThingSpeak based external monitoring at a total cost of USD 43.

**Table 10:** Comparison of the proposed system with existing IoT based poultry monitoring systems.

Ref.	Main monitored parameters	No. of parameters	Field validation	Cloud platform
[17]	Temperature, humidity, CO_2_, pressure	3–4	Short term, single house; limited days.	Local interface, no long-term cloud backend.
[26]	Temperature, humidity, ammonia	3	Prototype level testing, limited time.	Basic remote monitoring, no full cloud analytics.
[32]	Temperature, humidity, some air quality variables	3–4	Limited duration.	Custom IoT platform, no long-term data storage scenarios.
[38]	Temperature, fire/security status	2	Prototype model, no detailed long run.	SMS-based alerts only.
This Paper	Temperature, humidity, NH_3_, CH_4_, dust, light, litter moisture	7	15-day continuous field test in a commercial poultry farm.	ThingSpeak cloud with real-time remote visualization.

### 6.1. Cost analysis

The total cost of developing the prototype model is 43 USD. This is tolerable for any farmer who wants to develop their career in poultry farming. As many youths in Bangladesh are pursuing this field as a career, making this device cheaply is also an advantage for them. In developing countries, monitoring systems are often underdeveloped because devices are costly; our model costs less than 50 USD, making it affordable for any subcontinental country. A breakdown of total costs is given in Table 11.

**Table 11:** Cost analysis of the developed system.

Sl No	Components Name	Cost (BDT)
1	Esp32	400
2	BME 680	800
3	MQ-4	120
4	MQ-137	1000
5	BH 1750	200
6	Water Flow Sensor	300
7	Capacitive Soil Sensor	150
8	Dust Sensor	500
9	Buck Module	150
10	Mini Water Pump	400
11	Battery	500
12	Mini Solar Panel	400
13	OLED Display	150
14	Buzzer	10
15	Red Led	10
16	Fan	400
17	3D Box	100
	Total (BDT)	5190
	**Total (USD)**	**43**

### 6.2. Conclusion

In this research, a smart poultry farm monitoring device has been designed, implemented, and tested under practical farm conditions. The system was motivated by the limitations of many existing monitoring devices, which often show unsatisfactory accuracy and monitor only a narrow range of environmental parameters. In contrast, the proposed device integrates seven sensors within a single compact, low-cost platform and has undergone extensive prototype testing, including a 15-day field validation against commercially available reference instruments. The system provides early detection alerts, automated evaporative cooling control, solar powered operation, and cloud based remote monitoring, all at a total cost of USD 43, which makes it suitable for deployment in resource limited settings such as small and medium poultry farms in Bangladesh. Live alert functionality enables faster corrective actions without guesswork, while automation reduces human error and lowers the labor required for routine environmental checks. By maintaining more stable environmental conditions, the system helps to reduce stress, keep bird’s cleaner, and lower the risk of disease spread within the flock. Future enhancements may include the integration of mobile application alerts and additional remote tracking features to further increase usability and robustness in diverse farm contexts.

## Supporting information

S1 Fig Graphical Abstract. (TIF)

S1 File Source code and demonstration video of the IoT-based poultry farm monitoring system. Compressed folder containing (1) the complete ESP32 source code for sensor acquisition, actuator control, water flow monitoring, OLED display, and ThingSpeak communication, and (2) a demonstration video showing the system’s operation. Also Available at: https://github.com/mdzahidhasanbuiyan/IoT-Based-Automated-Poultry-Farming-Monitoring?tab=readme-ov-file
(ZIP)

## Abbreviations

IoT: Internet of Things
FCR: Feed Conversion Ratio
PPM: Parts Per Million
CAGR: Compound Annual Growth Rate
RH: Relative Humidity
SEM: Standard Error of Mean
SD: Standard Deviation
LED: Light Emitting Diode
NH_3_: Ammonia
CH_4_: Methane
BME680: Bosch Multi-Environment Sensor
MQ137: Metal Oxide gas sensor for Ammonia
MQ4: Metal Oxide gas sensor for Methane
BH1750: Digital light intensity sensor
OLED: Organic Light Emitting Diode
BDT: Bangladeshi Taka
USD: United States Dollar
HPAI: Highly Pathogenic Avian Influenza.
